# Obstructive Sleep Apnoea in Stanford Type B Aortic Dissection Is Associated With Multiple Imaging Signs Related to Late Aortic Events

**DOI:** 10.3389/fcvm.2021.752763

**Published:** 2021-11-18

**Authors:** Jiawei Zhang, Zhe Zhang, Lingyu Fu, Lei Wang, Yu Yang, Hao Wang, Baosen Zhou, Wei Wang, Jian Zhang, Shijie Xin

**Affiliations:** ^1^Department of Vascular Surgery, The First Hospital of China Medical University, Shenyang, China; ^2^Department of Clinical Epidemiology and Evidence Medicine, The First Hospital of China Medical University, Shenyang, China; ^3^Department of Vascular Surgery, Beijing Friendship Hospital, Capital Medical University, Beijing, China; ^4^Department of Respiratory Medicine, The First Hospital of China Medical University, Shenyang, China

**Keywords:** Stanford type B aortic dissection, obstructive sleep apnoea (OSA), late aortic events, aortic morphological changes, aortic dilatation

## Abstract

**Background:** Obstructive sleep apnoea (OSA) is highly prevalent in patients with Stanford type B aortic dissection (TBAD). Few studies have evaluated the effects of OSA on vascular changes in TBAD patients. This study aimed to explore the effect of OSA on aortic morphological changes in TBAD patients and its relation to late aortic events (LAEs).

**Methods:** This case-control study included 143 TBAD patients. The diameters of different parts of the aorta were measured based on computed tomography angiography (CTA). According to the apnoea-hypopnoea index (AHI), OSA was classified as mild (5 ≤ AHI ≤ 15), moderate (15 < AHI ≤ 30), or severe (AHI > 30). The false lumen (FL) status was evaluated and classified as partially thrombosed, patent, or completely thrombosed.

**Results:** The OSA prevalence in TBAD patients was 64.3%, and image differences related to LAEs between TBAD patients with and without OSA included the maximum aortic diameter at onset (37.3 ± 3.9 vs. 40.3 ± 4.5 mm, *p* < 0.001), the FL diameter of the proximal descending thoracic aorta (16.0 ± 6.8 vs. 20.3 ± 4.7 mm, *p* < 0.001), and the proportion of the FL that was partially thrombosed (39.2 vs. 64.1%, *p* = 0.004). Additionally, in the multivariable analysis of patients with OSA, the risks of an aortic diameter ≥40 mm, a proximal descending aorta FL ≥ 22 mm and a partially thrombosed FL were 4.611 (95% CI: 1.796–11.838, *p* = 0.001), 2.544 (95% CI: 1.050–6.165, *p* = 0.039), and 2.565 (95% CI: 1.167–5.637, *p* = 0.019), respectively, after adjustment for confounding factors. Trend tests showed that the risks of an aortic diameter ≥40 mm and a partially thrombosed FL increased with increasing OSA severity.

**Conclusions:** TBAD patients with moderate to severe OSA have aortic dilatation in different parts of the aorta. OSA is an independent risk factor for multiple imaging signs related to LAEs, suggesting that OSA is an important factor affecting the prognosis of TBAD patients.

## Introduction

Stanford type B aortic dissection (TBAD) is a catastrophic vascular disease with high mortality and poor prognosis (1, 2) that is characterized by the blood passing through the first one-third of the external media in the aortic wall (3). The annual incidence of AD is estimated to be 2.6–3.6 per 100,000 adults worldwide (4–6). Most patients die within the first week, and ~25–50% of patients who survive the acute phase require surgery during the chronic phase (7, 8). The overall in-hospital mortality of TBAD was reported to be 13%. Despite the increase in the use of thoracic endovascular aortic repair (TEVAR) and the application of hybrid surgery, in-hospital mortality has not changed significantly in the past 20 years (9). Compared with medical therapy, TEVAR has proven to be more effective in improving aortic remodeling and long-term survival (10). However, neither TEVAR nor medical therapy can fully prevent aortic wall degeneration or disease progression. Approximately 35% of patients treated with TEVAR and 65% of patients receiving medical therapy develop late aortic events (LAEs) within 5 years (11, 12).

The occurrence of LAEs severely affects the prognosis of patients with TBAD (13, 14). In most studies, LAEs were defined as the development of aortic expansion (>55 mm), rapid dilatation of the dissected aorta (>10 mm per 10–12 mo), new dissection, malperfusion, rupture, or impending rupture and aortic-related death after patients were discharged following surgery or medical treatment (10, 15–19). Previous studies have demonstrated that several predictors could be used to identify TBAD patients at high risk for aortic expansion during follow-up and predict the individual risk of LAEs. Radiologic predictors included an initial maximum diameter of the aorta ≥40 mm (10, 15, 16, 20–23), a proximal descending thoracic aortic false lumen (FL) diameter ≥22 mm (24), a partially thrombosed FL (17, 25–28), a large entry tear >10 mm (10), a fusiform index ≥0.64 (29), and ulcer-like projections (30, 31). Clinical predictors included younger age (<60 years) (32, 33), heart rate ≥60/min (34), and Marfan syndrome (35). Laboratory findings involved fibrinogen-fibrin degradation product levels ≥20 mg/ml at admission (36) and peak C-reactive protein levels ≥9.61 mg/dl (37). Identification of these predictors might benefit patients through early TEVAR or closer follow-up and contribute to the development of individualized treatment strategies (14, 19, 38).

Obstructive sleep apnoea (OSA) is characterized by recurrent upper airway obstruction with interruption of airflow and persistence of inspiratory effort during sleep (39) and is one of the most common risk factors for cardiovascular diseases (40). The prevalence of OSA in TBAD is 66.2–81.7% (41–43), which is dramatically higher than the reported incidence of 9–38% in the general population (44, 45). The potential correlation between OSA and TBAD was first reported by Sampol et al. (46). An increasing number of studies have shown that OSA is an important cause of AD (42, 47–49).

However, few studies have evaluated the effects of OSA on vascular changes in TBAD patients. The aim of this study was to explore the effect of OSA on aortic morphological changes in TBAD patients and its relation to LAEs.

## Materials and Methods

### Study Population

**Figure 1:**
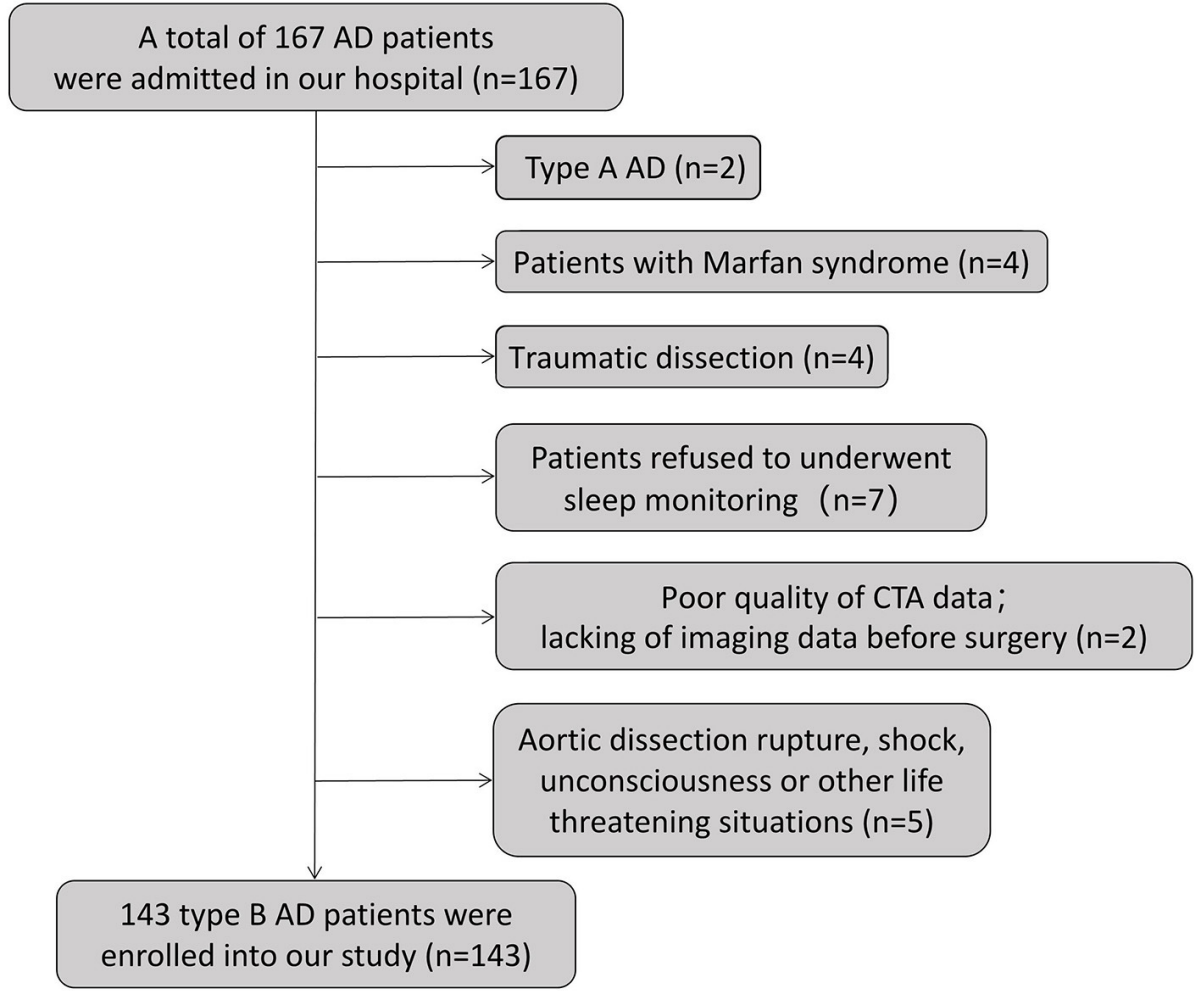
Flow chart of the exclusion criteria.

From August 1, 2019, to July 1, 2021, a total of 167 TBAD patients were admitted to the vascular surgery department of the First Hospital of China Medical University. All patients underwent computed tomography angiography (CTA) examinations and arterial blood gas analysis on admission. We routinely performed sleep monitoring on the patients. We asked the patients' medical history and queried their electronic medical records to determine whether the patients had undergone polysomnography (PSG) for the first time or had been previously treated with continuous positive airway pressure (CPAP) for OSA or other respiratory diseases. Smoking history was determined as a history of smoking (current or former) or no history of smoking. Figure 1 illustrates the exclusion criteria for this study, including (1) type A AD; (2) patients with Marfan syndrome; (3) patients with traumatic dissection; (4) patients who refused to undergo sleep monitoring; (5) poor-quality CTA data or a lack of imaging data before surgery; and (6) aortic dissection rupture, shock, unconsciousness or other life-threatening situations. A total of 143 TBAD patients were ultimately enrolled in our study.

### Imaging Data

CTA images were produced by a SIEMENS SOMATOM Definition Flash CT (Siemens Healthcare, Erlangen, Germany). The thickness of the CTA imaging slice was 1 mm. The aortic morphology was measured using image processing software (IMPAX Client, Agfa HealthCare N.V., Version 6.5.3.1509). The imaging data we measured included (1) the maximum aortic diameter at onset (the first CTA scan was performed during the acute event on initial imaging of the aorta); (2) the maximum diameter of the descending aorta; (3) the FL diameter of the proximal descending thoracic aorta (the FL at the upper descending thoracic aorta on initial CTA); (4) the diameter of the distal aortic arch (at the level of the left subclavian artery, including the retrograde dissection extending to the proximal aortic arch); (5) the diameter of the descending aorta (at the level of origin, the main pulmonary artery); (6) the maximum diameter of the FL; (7) the FL status (completely thrombosed, partially thrombosed, patent); (8) the fusiform index (defined as A/(B+C), where A = maximum diameter of the descending aorta, B = diameter of the distal aortic arch, C = diameter of the descending aorta at the level of origin, the main pulmonary artery) (29); and (9) the number of dissection process-involved zones according to the Society for Vascular Surgery/Society of Thoracic Surgeons (SVS/STS) classification system reporting standards for TBAD (50).

### Sleep Study

A sleep study was performed with a portable sleep-respiration monitor [PSM, PSM100A; Sealand Technology (Chengdu) Co., Ltd]. Sleep monitoring was performed before surgery and after all the patients were transferred from the intensive care unit to the general ward. The initial symptoms of each patient were relieved, which did not affect sleep monitoring due to pain or dyspnoea. Neither sedatives nor alcohol was used within 48 h before the study. The recording duration was between 10:00 P.M. to 7:00 A.M. and lasted at least 7 h. The content of the records included the apnoea-hypopnoea index (AHI), nasal airflow pressure, chest movement, body position, snoring and fingertip oximetry. The diagnostic criteria for OSA refer to the American Academy of Sleep Medicine (AASM) Guidelines (2017) (51) and were defined as AHI ≥ 5 events/h. OSA severity was categorized according to the AHI as follows: mild OSA (5 ≤ AHI ≤ 15), moderate OSA (15 < AHI ≤ 30), and severe OSA (AHI > 30).

### Statistics

All data are expressed as the mean ± standard deviation (SD) or number of participants (percentages). Normality was assessed using the Shapiro-Wilk normality test. Student's *t*-test was used for comparisons between TBAD patients with or without OSA if the data fit the normal distribution, and the Mann–Whitney *U*-test otherwise. Pearson's chi-square test or Fisher's exact test was used to compare the percentages in different groups. Univariate and multivariate logistical regressions were performed to analyse the risk factors and risk magnitude for a maximum aortic diameter at onset ≥40 mm, a diameter of the FL of the proximal descending thoracic aorta ≥22 mm and a partially thrombosed FL. Variables with a *p* ≤ 0.05 on univariate analysis and clinically important factors [age, hypertension, and body mass index (BMI)] were included in the multivariate analysis. The results are expressed as odds ratios (ORs) and 95% confidence intervals (CIs). *P* ≤ 0.05 were considered significant. Statistical analyses were performed using SPSS version 26 (IBM Corp.). The minimal sample size was estimated by PASS 11 (NCSS, Kaysville, Utah) and met the requirements of our study (for a maximum aortic diameter at onset ≥40 mm, a FL diameter of the proximal descending aorta ≥22 mm, and a partially thrombosed FL, the minimum sample size of TBAD patients with and without OSA was 58 and 32, 74 and 41, 87 and 48, respectively).

## Results

### Baseline Characteristics and Prevalence of OSA Among TBAD Patients

Of all 143 patients, the mean age was 52.0 ± 11.7 (years), and the average AHI was 15.1 ± 15.5 (times/h). Most of the patients underwent sleep monitoring for the first time, except for two patients who were previously diagnosed with OSA. One of the two patients received surgical treatment for OSA, and none of them had received CPAP. However, the AHI of the two patients was still as high as 28.5 and 22.2 times/h, respectively. Therefore, we still included the two patients in the trial group. A total of 71 patients (49.7%) had a pO_2_ <80 mmHg, of which seven patients (4.9%) had a pO_2_ <60 mmHg. In addition, the pCO_2_ and lactate levels of all the patients were within the normal range at admission. According to the latest SVS/STS classification system, we also counted the number of dissection process-involved zones. The clinical characteristics and imaging data of all the patients are shown in Table 1.

**Table 1:** Baseline characteristics of the patients.

**Variable**	**No. or mean (%)**
**Demographic characteristics**	
Male	115 (80.4)
Age, years	52.0 ± 11.7
Young adults (>18, ≤45)	37 (25.9)
Middle-aged adults (>45, <60)	68 (47.6)
Older adults (≥60)	38 (26.6)
Patients with OSA	92 (64.3)
Mild OSA	38(26.6)
Moderate OSA	27(18.9)
Severe OSA	27(18.9)
BMI (kg/m^2^)	26.8 ± 4.1
Hypertension	107 (74.8)
Diabetes	5 (3.5)
History of cardiovascular disease	8 (5.6)
Smoking	71 (49.7)
AHI (times/h)	15.1 ± 15.5
**Arterial blood gas analysis**	
PH	7.42 ± 0.03
pCO_2_ (mmHg)	38.7 ± 3.4
pO_2_ (mmHg)	82.0 ± 15.3
Lactate (mmol/L)	1.37 ± 0.56
**Contrast-enhanced CT data**	
Maximum aortic diameter at onset (mm)	39.2 ± 4.5
≥40 mm	63 (44.1)
<40 mm	80 (55.9)
Maximum diameter of the descending aorta (mm)	39.7 ± 4.8
FL diameter of the proximal descending aorta (mm)	18.8 ± 5.9
≥22 mm	50 (35.5)
<22 mm	91 (64.5)
Diameter of the distal aortic arch (mm)	31.5 ± 3.9
Diameter of the descending aorta at the level of the main pulmonary artery (mm)	34.6 ± 4.7
Maximum diameter of FL (mm)	20.6 ± 6.0
**False lumen status**	
Partially thrombosed	79 (55.2)
Patent	37 (25.9)
Completely thrombosed	27 (18.9)
Fusiform index	0.60 ± 0.04
≥0.64	22 (15.4)
<0.64	121 (85.6)
Numbers of dissection process-involved zones	7.8 ± 2.0

The prevalence of OSA in TBAD patients was 64.3%: 38 patients (26.6%) had mild OSA, 27 (18.9%) had moderate OSA, and 27 (18.9%) had severe OSA. TBAD patients with OSA showed a higher BMI of 28.0 ± 4.1 kg/m^2^ (p < 0.001) and a series of imaging differences. The main manifestation was aortic dilatation, which included the maximum aortic diameter at onset (37.3 ± 3.9 vs. 40.3 ± 4.5 mm, *p* < 0.001), the proximal descending thoracic aorta FL diameter (16.0 ± 6.8 vs. 20.3 ± 4.7 mm, *p* < 0.001), and the proportion of FL that was partially thrombosed (39.2 vs. 64.1%, *p* = 0.004). Other imaging differences included the maximum diameter of the descending aorta (38.2 ± 4.9 vs. 40.5 ± 4.5 mm, *p* = 0.029), the diameter of the distal aortic arch (30.4 ± 3.4 vs. 31.9 ± 3.3 mm, *p* = 0.008), and the maximum diameter of the FL (18.8 ± 7.5 vs. 21.6 ± 4.7 mm, *p* = 0.018). Two of the patients' FL were found in the abdominal aorta; therefore, we excluded these patients when analyzing the association between OSA status and the diameter of the FL of the proximal descending aorta. The characteristics of the TBAD patients with and without OSA are shown in Table 2.

**Table 2:** Characteristics of the TBAD patients with and without OSA.

**Variable**	**Patients without OSA** **(*n* = 51)**	**Patients with OSA** **(*n* = 92)**	***p*-value**
Male, *n* (%)	39 (76.5)	76 (82.6)	0.38
Age, years	51.4 ± 11.2	52.3 ± 12.1	0.67
BMI (kg/m^2^)	24.7 ± 3.2	28.0 ± 4.1	<0.001
Hypertension, *n* (%)	37 (72.5)	70 (76.1)	0.64
Smoking, *n* (%)	26 (51.0)	45 (48.9)	0.81
AHI (times/h)	2.8 ± 1.4	22.0 ± 15.5	<0.001
Maximum aortic diameter at onset (mm)	37.3 ± 3.9	40.3 ± 4.5	<0.001
Maximum aortic diameter at onset ≥40 mm, *n* (%)	13 (25.5)	50 (54.3)	<0.001
Maximum diameter of the descending aorta (mm)	38.2 ± 4.9	40.5 ± 4.5	0.029^*^
FL diameter of the proximal descending thoracic aorta (mm)	16.0 ± 6.8	20.3 ± 4.7	<0.001
FL diameter of the proximal descending thoracic aorta ≥22 mm, *n* (%)	11 (21.6)	39 (42.4)	0.012
Diameter of the distal aortic arch (mm)	30.4 ± 3.4	31.9 ± 3.3	0.008
Diameter of the descending aorta at the level of the main pulmonary artery (mm)	33.7 ± 5.3	35.0 ± 4.3	0.045^*^
Maximum diameter of the FL (mm)	18.8 ± 7.5	21.6 ± 4.7	0.018
Fusiform index	0.597 ± 0.04	0.600 ± 0.04	0.287
Fusiform index ≥0.64, *n* (%)	6 (11.8)	16 (17.4)	0.372
Partially thrombosed, *n* (%)	20 (39.2)	59 (64.1)	0.004
Numbers of dissection process-involved zones	7.4 ± 2.2	8.1 ± 1.8	0.065^*^

* *This variable was analyzed with the Mann–Whitney U-test*.

### Association Between OSA Status and the Maximum Aortic Diameter at Onset

**Figure 2:**
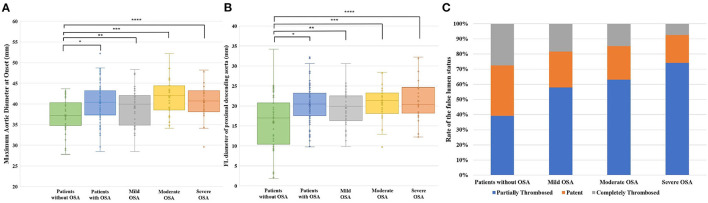
Imaging signs related to LAEs in different OSA severities. **(A)** Aortic diameter at onset for different degrees of OSA severity. *p < 0.001; **p = 0.044; ***p < 0.001; ****p = 0.001. **(B)** FL diameter of the proximal descending thoracic aorta for different OSA severities. *p < 0.001; **p = 0.004; ***p < 0.001; ****p < 0.001. **(C)** Rate of the FL status in different OSA severities.

As shown in Figure 2A, compared with patients without OSA, patients with OSA showed larger aortic diameters. Table 3 displays the results for the univariate and multivariate logistic regression analyses on a maximum aortic diameter at onset ≥40 mm. The risk of an aortic diameter larger than 40 mm in TBAD patients with OSA was ~4 times higher (OR: 4.225, 95% CI: 1.962–9.099, *p* < 0.001) than that in patients without OSA. After adjusting for age and hypertension (model 1) and for age, hypertension, sex, and BMI (model 2), the ORs were 5.689 (95% CI: 2.361–13.704, *p* < 0.001) and 4.611 (95% CI: 1.796–11.838, *p* = 0.001), respectively. We also analyzed the effect of different OSA severities on the aortic diameter, as shown in Table 4. After adjusting for all the relevant factors (model 2), the ORs for patients with mild, moderate and severe OSA were 4.189 (95% CI: 1.454–12.071, *p* = 0.008), 6.684 (95% CI: 1.933–23.107, *p* = 0.003), and 4.830 (95% CI: 1.387–16.824, *p* = 0.013), respectively. Trend analysis showed that the risk of an aortic diameter ≥40 mm increased with increasing OSA severity (*p* = 0.006; Table 4).

**Table 3:** Univariate and multivariate logistic analyses of imaging signs related to LAEs.

**Variables**	**Univariate analysis**		**Multivariate (Model 1)**		**Multivariate (Model 2)**	
	**OR** **(95% CI)**	***p*-value**	**OR** **(95% CI)**	***p*-value**	**OR** **(95% CI)**	***p*-value**
**Maximum aortic diameter at onset** **≥40 mm**						
Age	1.081 (1.044–1.120)	<0.001	1.084 (1.041–1.128)	<0.001	1.097 (1.047–1.149)	<0.001
Sex	0.643 (0.281–1.475)	0.298	–	–	0.894 (0.306–2.612)	0.838
BMI	1.030 (0.951–1.116)	0.469	–	–	1.074 (0.956–1.206)	0.228
OSA	4.225 (1.962–9.099)	<0.001	5.689 (2.361–13.704)	<0.001	4.611 (1.796–11.838)	0.001
Hypertension	4.723 (1.904–11.716)	0.001	4.078 (1.457–11.420)	0.007	3.646 (1.278–10.401)	0.016
**FL diameter of the proximal descending thoracic aorta** **≥22 mm**						
Age	1.008 (0.978–1.038)	0.605	–	–	1.007 (0.970–1.044)	0.730
Sex	1.113 (0.460–2.690)	0.813	–	–	1.160 (0.448–3.003)	0.760
BMI	1.071 (0.983–1.167)	0.118	1.024 (0.932–1.125)	0.617	1.032 (0.926–1.151)	0.568
OSA	2.868 (1.278–6.437)	0.011	2.626 (1.102–6.257)	0.029	2.544 (1.050–6.165)	0.039
Hypertension	2.523 (1.010–6.305)	0.048	2.440 (0.958–6.219)	0.062	2.381 (0.899–6.311)	0.081
**Partially thrombosed FL**						
Age	1.005 (0.977–1.034)	0.724	–	–	1.002 (0.969–1.037)	0.886
Sex	0.909 (0.395–2.092)	0.822	–	–	0.826 (0.341–2.002)	0.672
BMI	1.067 (0.982–1.159)	0.126	1.021 (0.932–1.117)	0.660	1.027 (0.927–1.137)	0.616
OSA	2.771 (1.369–5.610)	0.005	2.583 (1.201–5.557)	0.015	2.565 (1.167–5.637)	0.019
Hypertension	1.790 (0.835–3.834)	0.134	1.762 (0.803–3.865)	0.158	1.723 (0.767–3.870)	0.188

**Table 4:** Logistic regression and trend analyses of imaging signs related to LAEs for different OSA severities.

**OSA severity classification**	**OR (95% CI)** ***p*****-value**		
	**Non-adjusted**	Model 1^*^	Model 2^**^
**Maximum aortic diameter at onset** **≥40 mm**			
Non-OSA	Reference	Reference	Reference
Mild OSA	5.381 (2.083–13.902) 0.001	4.665 (1.650–13.191) 0.004	4.189 (1.454–12.071) 0.008
Moderate OSA	7.380 (2.616–20.817) <0.001	8.157 (2.469–26.941) 0.001	6.684 (1.933–23.107) 0.003
Severe OSA	5.125 (1.836–14.308) 0.002	6.489 (2.056–20.481) 0.001	4.830 (1.387–16.824) 0.013
*P* for trend	<0.001	<0.001	0.006
**FL diameter of the proximal descending thoracic aorta** ≥**22 mm**			
Non-OSA	Reference	Reference	Reference
Mild OSA	3.106 (1.171–8.237) 0.023	2.810 (1.042–7.574) 0.041	2.792 (1.019–7.646) 0.046
Moderate OSA	3.644 (1.279–10.386) 0.016	3.301 (1.121–9.719) 0.030	3.207 (1.050–9.793) 0.041
Severe OSA	3.644 (1.279–10.386) 0.016	2.733 (0.867–8.620) 0.086	2.669 (0.835–8.530) 0.098
*P* for trend	0.011	0.060	0.079
**Partially thrombosed FL**			
Non-OSA	Reference	Reference	Reference
Mild OSA	2.131 (0.907–5.010) 0.083	2.111 (0.877–5.081) 0.096	2.067 (0.846–5.052) 0.111
Moderate OSA	2.635 (1.007–6.898) 0.048	2.763 (1.006–7.591) 0.049	2.775 (0.971–7.933) 0.057
Severe OSA	4.429 (1.584–12.380) 0.005	4.207 (1.286–13.766) 0.018	4.228 (1.282–13.945) 0.018
*P* for trend	0.003	0.010	0.011

* *For the maximum aortic diameter at onset ≥40 mm, the model was adjusted for age and hypertension. For the FL diameter of the proximal descending thoracic aorta ≥22 mm and partially thrombosed FL, the model was adjusted for BMI and hypertension*;

** *Adjusted for age, hypertension, sex, and BMI*.

### Association Between OSA Status and the FL Diameter of the Proximal Descending Aorta

TBAD patients with OSA showed a larger proximal descending aorta FL diameter (Table 2). As OSA severity increased, the FL diameter also increased gradually (Figure 2B), but for a proximal descending aorta FL ≥ 22 mm in different OSA severities, as shown in Table 4, a trend analysis did not show a significant increase in OSA severity (*p* = 0.079). Univariate logistic regression showed that age, sex, and BMI were not risk factors for dilatation of the proximal descending aorta FL (≥22 mm). However, OSA was a significant risk factor (OR: 2.868, 95% CI: 1.278–6.437, *p* = 0.011) for FL dilatation. After adjusting for all the relevant factors (age, hypertension, sex, and BMI), the OR was 2.544 (95% CI: 1.050–6.165, *p* = 0.039; Table 3).

### Association Between OSA Status and Thrombosed FL

Most of the patients had partially thrombosed FLs. However, the proportion of partially thrombosed FLs in severe OSA patients was as high as 74.1%, which was much higher than that in patients without OSA (39.2%) (*p* = 0.004, χ^2^ = 8.238). The proportions of patients with mild and moderate OSA were 57.9 and 63.0%, respectively (Table 5). Univariate and multivariate logistic regression analyses on partially thrombosed FL are shown in Table 3. After adjusting for confounding factors, the ORs of OSA for model 1 and model 2 were 2.583 (95% CI: 1.201–5.557, *p* = 0.015) and 2.565 (95% CI: 1.167–5.637, *p* = 0.019), respectively. Figure 2C shows the rate of the FL status in different OSA severities. Trend analysis showed that the risk of a partially thrombosed FL increased with increasing OSA severity (*p* = 0.011; Table 4).

**Table 5:** Thrombosed FL with different OSA severity levels.

**Variable**	**Patients without OSA** **(*n* = 51)**	**Patients with OSA**			***p*-value**	**χ^2^**
		**Mild OSA** **(*n* = 38)**	**Moderate OSA** **(*n* = 27)**	**Severe OSA** **(*n* = 27)**		
Partially thrombosed	20 (39.2%)	22 (57.9%)	17 (63.0%)	20 (74.1%)	0.004	8.238
Patent	17 (33.3%)	9 (23.7%)	6 (22.2%)	5 (18.5%)	0.129	2.300
Completely thrombosed	14 (27.5%)	7 (18.4%)	4 (14.8%)	2 (7.4%)	0.051	3.801

### Other Imaging Differences Between TBAD Patients With and Without OSA

**Figure 3:**
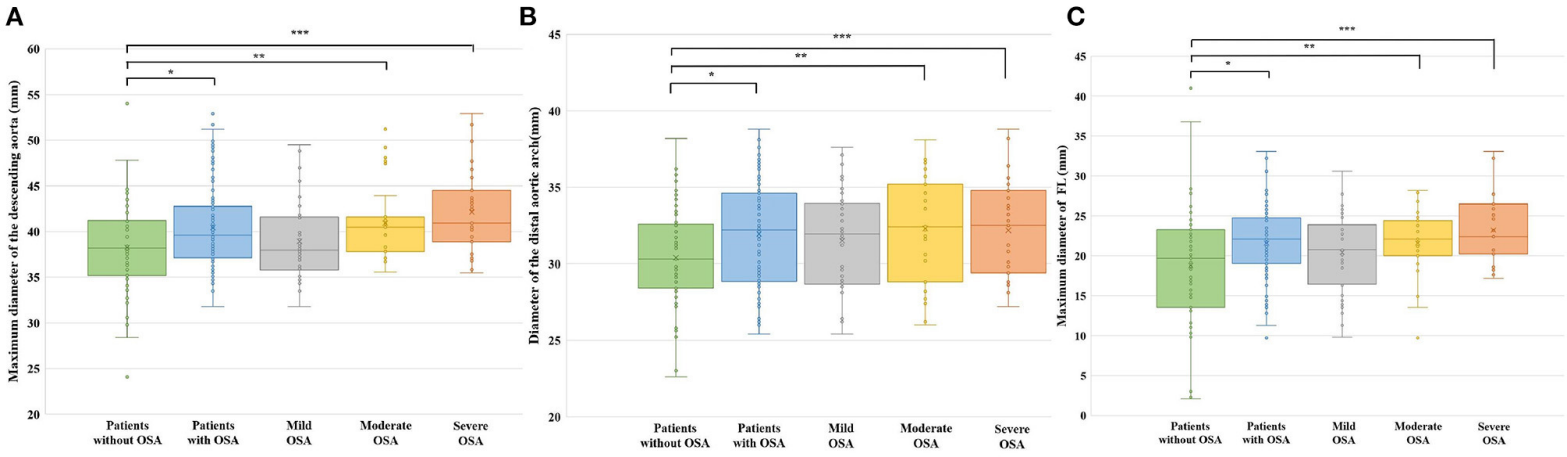
Other imaging differences in different OSA severities. **(A)** Maximum diameter of the descending aorta for different OSA severities. *p = 0.029; **p = 0.037; ***p = 0.001. **(B)** Diameter of the distal aortic arch for different OSA severity levels. *p = 0.008; **p = 0.022; ***p = 0.026. **(C)** Maximum diameter of the FL for different OSA severity levels. *p = 0.018; **p = 0.038; ***p = 0.006.

As shown in Figure 3, other significant imaging differences between the two groups included the maximum diameter of the descending aorta (38.2 ± 4.9 vs. 40.5 ± 4.5 mm, *p* = 0.029), the diameter of the distal aortic arch (30.4 ± 3.4 vs. 31.9 ± 3.3 mm, *p* = 0.008), and the maximum diameter of the FL (18.8 ± 7.5 vs. 21.6 ± 4.7 mm, *p* = 0.018). We further analyzed these imaging differences according to OSA severity and found that the most significant difference was between non-OSA patients and patients with moderate and severe OSA.

## Discussion

To our knowledge, this is the first study to find that TBAD patients with OSA have a higher risk of aortic dilatation and that some of the imaging differences are related to LAEs.

This case-control study included 143 TBAD patients, and the prevalence of OSA was as high as 64.3%. In 2003, Sampol et al. (46) first found a correlation between OSA and aortic dissection, and an increasing number of studies have shown that OSA is an independent risk factor for AD, especially for TBAD patients with moderate to severe OSA (47–49). Previous studies have indicated that OSA may lead to dilatation of the aorta, including the aortic root (52–55), thoracic aorta (56) and abdominal aorta (57); other studies have come to the opposite conclusion (58), and most of the OSA patients in these studies had no history of cardiovascular disease. However, the effect of OSA on vascular morphology changes in TBAD has rarely been reported. As seen in our results, the main change in the aorta for TBAD patients with OSA is aortic dilatation, which also includes enlargement of the FL. The mechanism of aortic dilatation and the development of TBAD induced by OSA has not been fully elucidated. The possible mechanism includes changes in intrathoracic pressure, meaning that the increase in pleural negative pressure can lead to mechanical stretch of the aorta (54, 55, 59–61), which further leads to the expansion of the aortic root, thoracic aorta and thoracic FL; patients with frequent intermittent hypoxia leading to oxidative stress and increases in sympathetic activity exhibit an increase in blood pressure due to the activation of the renin–angiotensin–aldosterone system (59, 60, 62). The state of chronic intermittent hypoxemia and the altered microenvironment in OSA patients, such as the increase in IL-6 levels and reduction in TGF-β levels, could damage the function of endothelial cells and promote a Th17/Treg imbalance, which leads to a reduction in the production of endothelium-dependent vasodilator substances and contributes to vascular dysfunction and stimulation of systemic inflammation (63–67).

Another important finding in our study showed that vascular changes in TBAD patients with OSA were related to the predictors of LAEs, which included an initial aortic diameter ≥40 mm, a FL diameter of the proximal descending thoracic aortic ≥22 mm, and a partially thrombosed FL. Previous studies have proven that the postdischarge mortality of patients with these factors will be substantially increased (10, 15, 20, 21, 23–25). An aortic diameter >40 mm and a FL diameter >22 mm have been suggested as high-risk features by SVS/STS research (50). Our results indicate that for patients with OSA, the risk of these imaging signs was several times higher than that of non-OSA patients. Univariate analysis revealed that the risk of aortic diameter dilation larger than 40 mm in OSA patients was ~4.6 times higher than that in non-OSA patients when we adjusted for age, sex, BMI, and hypertension in the multivariate analysis. In addition, age is an independent risk factor for aortic enlargement in TBAD patients, which suggests that the degree of aortic dilatation is more obvious in elderly TBAD patients with OSA.

In addition, we observed the effect of OSA on the size and thrombosis of the FL. Song et al. (24) revealed that patients with an FL diameter of the initial upper descending thoracic aorta ≥22 mm showed a higher rate of aortic adverse events such as aneurysmal dilation or aortic-related death. From the results, the risk of a proximal descending aortic FL larger than 22 mm was ~2.5 times higher in OSA patients than in non-OSA patients after adjusting for other relevant factors (model 2). This suggests that OSA is an important factor related to the prognosis of TBAD patients. Our study shows that OSA is an important factor leading to an increased risk of partial FL thrombosis. Wang et al. (41) came to the same conclusion. The results showed that the risk of FL thrombosis in patients with OSA is increased by ~2.5-fold and that the proportion of FL thrombosis increases with the severity of OSA. Patients with OSA are in a state of chronic intermittent hypoxemia, which may lead to endothelial cell damage and the release of inflammatory factors, causing hypercoagulability of blood. This process increases the chance of FL thrombosis and is accompanied by intrathoracic pressure fluctuations, which leads to changes in aortic transmural pressure and mechanical stretching of the aorta. Furthermore, it may interfere with thrombosis of the FL, and both processes eventually cause a higher probability of partial thrombosis.

We classified OSA according to its severity as mild, moderate and severe. As seen in our results, moderate OSA patients showed a higher OR than severe and mild OSA patients in the logistic regression analyses on an aortic diameter ≥40 mm. This may be due to the sampling error caused by the relatively small sample size after grouping or other unknown factors in moderate OSA. Most of the vascular morphology differences were reflected in the patients with moderate and severe OSA, including the aortic diameter at onset, the maximum diameter of the descending aorta, the diameter of the distal aortic arch, and the maximum diameter of the FL. Moderate to severe OSA has a significant effect on morphological changes in the aorta. Furthermore, TBAD patients with moderate to severe OSA may have a worse prognosis.

We also measured other vascular morphological parameters correlated with prognosis that were reported by previous studies, including the fusiform index (29), but there was no significant difference between OSA and non-OSA in TBAD patients. This is because the fusiform index was defined to express the dilatation degree of the descending aorta, which is a method used to describe the morphological changes in the aorta by local dilation. However, our study found that patients with OSA tend to present dilation of the whole aorta, which may explain why there was no significant difference in the degree of local expansion between OSA and non-OSA patients. We used the SVS/STS classification system to describe the extent of FL involvement. There was no significant difference between OSA and non-OSA, which shows that although OSA can lead to enlargement of the FL, it cannot lead to extension of the FL. The SVS/STS classification system also provides a way for us to further study the area of dissection involved.

From the blood gas analysis results, nearly half of the patients had a decrease in pO_2_, of which 7 patients reached type I respiratory failure. We considered that this is an acute manifestation rather than a chronic progressive condition because when taking the patients' medical history, we found that almost all patients with decreased pO_2_ complained of dyspnoea and sweating caused by pain, and when we rechecked the blood gas before discharge, it had returned to the normal range.

The strengths of our study include that this is the first study to explore the effect of OSA on vascular morphological changes in TBAD patients and the first to identify OSA as an independent risk factor related to LAEs. To comprehensively and systematically assess changes in the aorta, we included up to nine indicators, including not only aortic dilatation, FL status, and imaging signs with LAEs but also the dissection process-involved zones according to the SVS/STS classification system. We first found that the effect of OSA on the aorta was mainly manifested as aortic dilation rather than an increase in the involved zones. There are still some limitations to our study, which include the need for further investigations in animal experiments to reveal the molecular mechanism of the occurrence and development of TBAD caused by OSA. Further studies on the prognosis of TBAD patients with OSA are also needed.

## Conclusion

TBAD patients with OSA, especially moderate to severe OSA, have vascular morphological changes in the different regions of the aorta that mainly manifest as aortic dilation. OSA was an independent risk factor for a maximum aortic diameter at onset ≥40 mm, an FL diameter in the proximal descending aorta ≥22 mm, and a partially thrombosed FL. The imaging differences related to LAEs suggest that OSA is an important factor that affects the prognosis of TBAD patients.

## Data Availability Statement

The original contributions presented in the study are included in the article/supplementary material, further inquiries can be directed to the corresponding author/s.

## Ethics Statement

The studies involving human participants were reviewed and approved by the Medical Research Ethics Committee of the First Hospital of China Medical University. The patients/participants provided their written informed consent to participate in this study.

## Author Contributions

JiawZ and SX first conceived this article, and designed the research method of this study. JiawZ was responsible for data analysis and writing the manuscript, collected the medical records, and imaging data as well as drafted the manuscript. ZZ and YY also collected the medical records and performed sleep monitoring. LW and HW drafted the discussion. LF and BZ gave the help of statistical analysis. WW and JianZ participated in the design of the study and analyzed sleep monitoring data. SX carried out critical revision and finalization of the manuscript. All authors approved the final manuscript.

## Funding

This work was supported by the National Natural and Science Foundation of China (Grant Numbers: 81974049).

## Conflict of Interest

The authors declare that the research was conducted in the absence of any commercial or financial relationships that could be construed as a potential conflict of interest.

## Publisher's Note

All claims expressed in this article are solely those of the authors and do not necessarily represent those of their affiliated organizations, or those of the publisher, the editors and the reviewers. Any product that may be evaluated in this article, or claim that may be made by its manufacturer, is not guaranteed or endorsed by the publisher.
